# Ocular Tuberculosis Without a Lung Primary

**DOI:** 10.7759/cureus.7920

**Published:** 2020-05-01

**Authors:** Nitish Singh Nandu, Aamani Bavanasi, Rana Wajahat

**Affiliations:** 1 Internal Medicine, Chicago Medical School, Rosalind Franklin University, North Chicago, USA; 2 Internal Medicine, MNR Medical College, Fasalwadi, IND; 3 Infectious Disease, Rosalind Franklin University, North Chicago, USA

**Keywords:** ocular tuberculosis, tuberculosis, disseminated tuberculosis

## Abstract

Tuberculosis (TB) is a multisystem infectious disease caused by *Mycobacterium tuberculosis*, which primarily affects the lungs. It is the leading infectious cause of morbidity and mortality worldwide with significant prevalence in the developing countries. However, extrapulmonary manifestations can be seldom seen in a few patients with disseminated TB or with localized disease. These manifestations depend on various comorbidities and the immune status of the patients. Extrapulmonary tuberculosis (EPTB) constitutes multiple cases of TB in the immunocompetent and immunocompromised populace. The clinical presentation of EPTB is atypical and can be challenging to confirm, often leading to delayed diagnosis and treatment. This, in particular, is true with ocular TB. The incidence of ocular TB is uncertain due to difficulties in ocular sampling for microbiology and the lack of definitive diagnostic criteria. Ocular TB can present in a fashion similar to other conditions causing ocular inflammation. It is crucial for physicians to consider this diagnosis in their differential, as ocular TB can present in a fashion similar to that of more common conditions causing ocular inflammation. We present a rare case of ocular uveitis secondary to TB in an asymptomatic patient without a lung primary, complicated by an unmasked allergy to first-line anti-TB medication.

## Introduction

Tuberculosis (TB) is an airborne communicable disease and a prevalent public health issue in developing countries. The incidence of TB is sporadic in the developed nations with infections commonly seen in immigrant workers, and travel to and from an endemic region. It is caused by *Mycobacterium tuberculosis* that primarily affects the lungs. Extrapulmonary manifestations can be seldom seen in a few patients with disseminated TB or with localized disease. Ocular TB is the manifestation of the infection in various tissues of the eye and includes choroiditis, uveitis, chorioretinitis, choroidal granuloma, optic neuritis, optic disc granuloma, subretinal abscess, orbital cellulitis, scleritis, necrotizing scleritis, posterior scleritis, sclerokeratouveitis, etc. [1]. The most common mode of transmission is hematogenous spread from pulmonary or extrapulmonary source. Infrequently ocular TB can occur as a result of direct ocular infection from an exogenous source. Uveitis can be seen concurrently with TB, but a direct association is difficult to prove. TB is frequently presumed in the presence of suggestive ocular findings in combination with systemic findings consistent such as positive acid-fast bacilli (AFB) smear and culture or consistent radiographic findings, or a positive interferon-gamma release assay (IGRA) or tuberculin skin test (TST) in individuals with no systemic symptoms [2]. We present such a rare case of ocular uveitis secondary to direct ocular infection from an exogenous source in an asymptomatic patient. His treatment course is complicated by an unmasked allergy to first-line anti-TB medication.

## Case presentation

**Figure 1:**
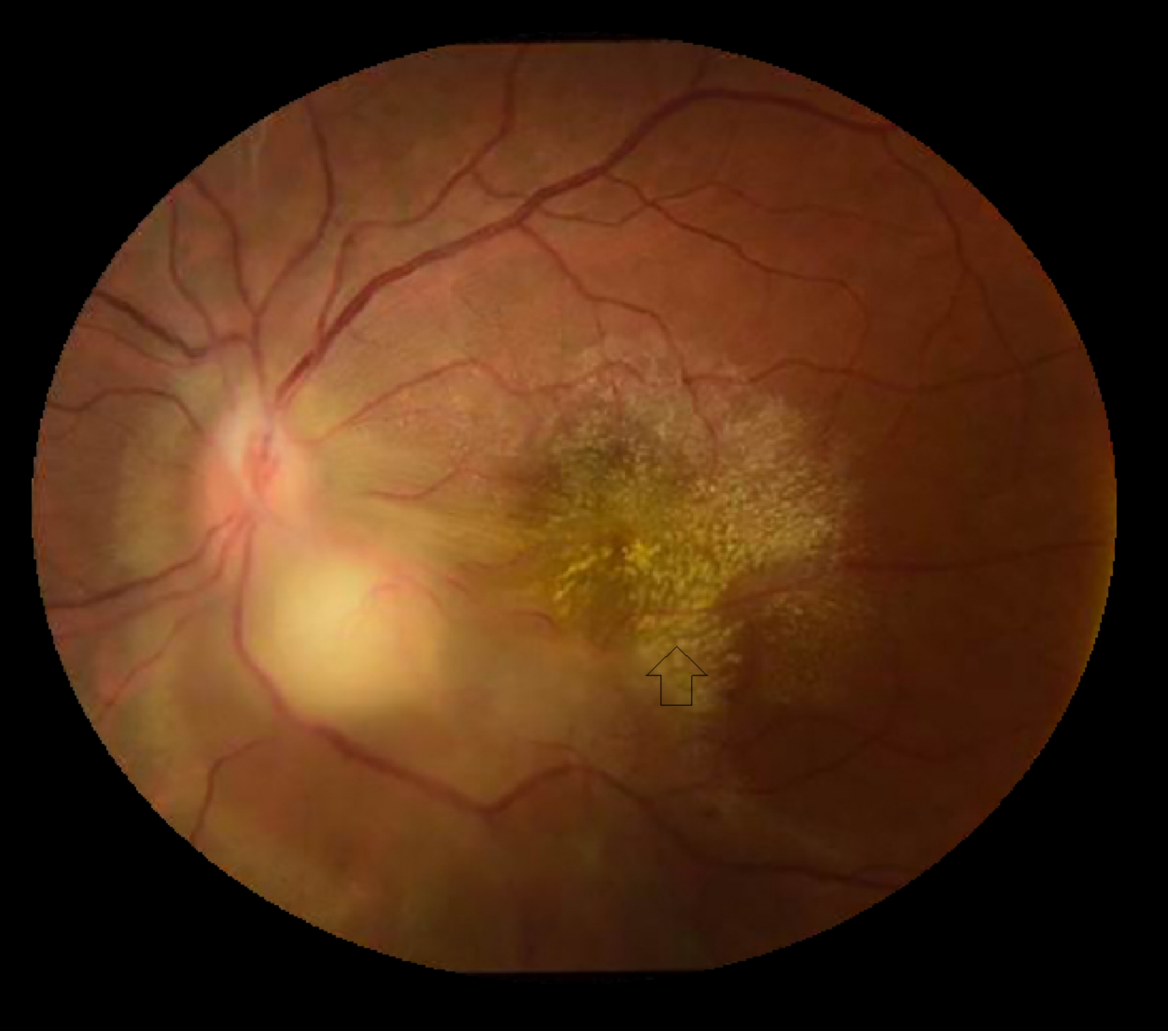
Ocular tuberculosis showing posterior uveitis with a granuloma in the right eye

A 39-year-old male patient with no significant past medical history presented with a chief complaint of blurry vision in the left eye. He reported no history of travel or recent sick contacts. He works as a welder and does not always use protective gear. He suspects he might have been exposed to TB from one of his coworkers whom he suspects may have had TB. The ophthalmic exam revealed panuveitis of the left eye (Figure 1). He complained of blurry vision in his left eye; however, he denied any chest pain, shortness of breath, hemoptysis, night sweats, or fevers. His physical exam was unremarkable. Labs were significant for a positive QuantiFERON (Qiagen, Hilden, Germany), and syphilis and HIV were negative. Chest X-ray was negative for infiltrates, cavities, or lymphadenopathy. A presumptive diagnosis of ocular TB was made, and the patient was started on isoniazid, ethambutol, pyrazinamide, rifampin, and vitamin B6 (RIPE regimen). He presented to the emergency department with a rash on the chest, back, and thigh three days after starting the anti-TB regimen. His labs at that time showed an elevation in the liver function test. He was recommended to stop all anti-TB regimens and follow up the following week. The patient however restarted the RIPE regimen after resolution of the rash and presented with a similar rash one week later. He was admitted to the medical floor for a drug provocation test. Anti-TB medications were started one by one in small doses and uptitrated. Isoniazid was started first, and the patient’s rash recurred two days later. The drug was discontinued, and he was given diphenhydramine for the rash and the drug rechallenge test was resumed with the rest of the TB medications. His liver function normalized after discontinuation of isoniazid, and he tolerated the rest of the regimen well. He was finally discharged on a full dose of pyrazinamide, ethambutol, and rifampin. During the one-month follow-up, he reported worsening vision and floaters in the right eye, ethambutol-induced optic neuropathy was suspected. and hence ethambutol was replaced with levofloxacin along with a short course of oral prednisone for suspected drug-induced optic neuropathy. The patient reported significant improvement in his vision, Ocular testing confirmed improvement in his vision (OD 20/70 from 20/200).

## Discussion

Ocular TB is usually not associated with clinical evidence of pulmonary tuberculosis, as up to 60% of extrapulmonary TB patients may not have an active pulmonary disease. The diagnosis of ocular TB can be challenging, and a definitive diagnosis requires isolation of bacilli from the ocular tissue that is difficult to achieve [3,4].* *Mycobacterial cultures require weeks to grow and have a low yield from the ocular samples [5]. Ocular TB can present in a fashion similar to other conditions causing ocular inflammation [6]. Hence, the diagnosis of TB is frequently presumed in the presence of suggestive ocular findings in combination with systemic findings consistent such as positive AFB smear and culture or consistent radiographic findings, or a positive IGRA or TST in individuals with no systemic symptoms [2]. Hence, it is a diagnosis of exclusion after ruling out syphilis and seronegative arthropathy. Ocular TB is treated with the same guidelines of active TB or extrapulmonary TB using standard anti-TB regimen with eight weeks of RIPE followed by 18 weeks of rifampin and isoniazid. Ocular involvement of TB may be superficial, intraocular, conjunctival, corneal, and scleral with or without systemic involvement. Posterior uveitis being the most common. This case was unique due to the rash secondary to treatment and using a protocol of drug provocation test challenge to identify the culprit drug. Anti-TB regimen drugs have various side effects and should be administered under close monitoring.

## Conclusions

Ocular TB is a rare phenomenon and is often a medical challenge to diagnose. The diagnosis is presumptive and the treatment protocol is similar to active TB elsewhere in the body. Anti-TB regimen drugs have various side effects and should be administered under close monitoring. Care should be taken prior to initiating the patient on antitubercular regimen, and these drugs need to be continued for multiple weeks with strict adherence to prevent resistance and prevent complications.
